# Identity suffering in infertile men

**DOI:** 10.1186/2051-4190-24-1

**Published:** 2014-01-02

**Authors:** Monique Jaoul, Marc Bailly, Martine Albert, Robert Wainer, Jacqueline Selva, Florence Boitrelle

**Affiliations:** Department of Reproductive Biology, Cytogenetics and Gynaecology, Poissy General Hospital, F-78303 Poissy, France; EA 2493, Versailles University of Medicine and Science, F-78000 Versailles, France

**Keywords:** Male infertility suffering, Objectal and identity suffering, Psycho-analytic approach, Souffrance de l’infertilité masculine, Souffrance objectale, Souffrance identitaire, Mécanismes de défense, Approche psychanalytique

## Abstract

The suffering caused by infertility in a man can have multiple aspects. It can display a *narcissistic* dimension, an *objectal* dimension (object-libido) turned toward others or/and an *identity* dimension. Two clinical case reports were used here to (i) illustrate all these aspects of infertility suffering, (ii) to evidence the difficulty for infertile men to speak about their infertility and (iii) underlie the importance for professional of medical assisted reproduction to be attentive to this suffering that many men keep silent. An empathetic attention to infertile men may give a way to express this suffering and thus allow the beginning of a psychoanalytic approach which is necessary in infertility and especially for infertile men who do not easily express their suffering.

## Resume

La souffrance de l’infertilité chez l’homme peut prendre plusieurs aspects. Elle peut avoir une dimension narcissique. Elle peut avoir une dimension objectale, être tournée vers l’autre. Elle peut avoir aussi une dimension identitaire particulièrement douloureuse car elle remet en cause l’identité sexuelle comme l’appartenance à la lignée. Deux cas cliniques vont éclairer cette souffrance que beaucoup d’hommes taisent d’une manière défensive.

Cette attitude défensive pourra donner le change et faire croire à une bonne adaptation à la situation. Malgré ou à cause de ce silence les acteurs de l’assistance médicale à la procréation devront rester attentifs à ne pas se laisser abuser par cette apparente absence de difficulté. Une attention empathique pourra donner l’occasion d’une expression de cette souffrance d’infertilité et ouvrir ainsi la possibilité d’une prise en charge psychothérapique jusque là inenvisageable.

## Introduction

Most studies on the psychological experience of infertility point out the depressive suffering common to men and women [1]. In infertile couples, psychological suffering could be expressed at various steps of the medical care: at the time of infertility announcement, during medical care and when medical care failed for example. Very often, the announcement of infertility causes the same reactions as that of mourning a death or a serious illness: it is revolt, jealousy, guilt, depression, anxiety and a desperate search for a cause. Furthermore, experimenting suicidal feelings and being strained in their marital, sexual and/or social relationships are not rare [2–4]. The path to a certain acceptance will vary in time and the way the medically assisted reproduction (MAR) process is experienced will depend on this acceptance. Infertility brings back past suffering: “the hope, the project of individual filiations always induces a subjective dream which reactivates the unconscious memory” said Bydlowski [5]. The suffering associated with the MAR process reinforces the suffering of infertility itself: it is a circular dynamic. According to Greil et al. [6], infertility treatment is associated with levels of distress over and above those associated with the state of being infertile in and of itself. The number of couples who give up the MAR program after a few attempts for psychological reasons is indeed significant even when the medical prognosis is encouraging [7, 8].

Research on infertility is much more focused on women experiences than in men [9, 10]. Women are more often psychologically affected than men by their infertility. They score higher on the questionnaire that evaluates stress and depression. A recent Danish cohort study [11] suggested an increased risk of psychiatric disorders in woman who do not give birth after MAR treatment. According to Pash et al. [12] to have a child is more important for women than for men. Women are indeed more involved in trying to have a baby and want to talk about trying to have a baby more often. This difference of perception may affect marital communication: wives perceive a positive effect of fertility on their marriage when husbands want to talk with their wives about trying having a baby. If we focus on infertile men, several studies showed that the feeling of infertility caused anxiety and could often lead to sexual dysfunctions because of the close relationship between fertility and sexuality [3, 13, 14]. Furthermore, family and friends of infertile men are rarely informed. On the contrary, when infertility is due to female causes, family and friends are frequently informed. So, all these studies reported difficulties for men to assume their infertility since fertility is confounded with sexuality and virility. Secondly, men often feel cast aside in the MAR process: they feel they are just asked to provide spermatozoa and experience guilt about what their partner must undergo, which reinforces their loss of their self-esteem [15, 16]. Globally, male partners in couples who feel that they are responsible for the couple infertility are at higher risk for sexual, emotional, and psychological strain relative to men without this belief [17]. However, it is important to notice that even when the announcement of their infertility causes them profound and lasting depressive reactions, men will have difficulties asking for psychological help [10]. Several studies conclude indeed that there are gender differences to cope with infertility and the trauma of procreation project failure [18, 19]. Men have more inhibited and controlled reactions. Their emotional difficulty is more often shown through a social and professional hyper activity rather than a frank depressive state. Some studies [20, 21] have dealt with psychic processes put in place to cope with the trauma of procreation project failure. They show reduced psychological and emotional functions from which conflicts and emotions were excluded. These elements reveal the traumatic dimension of both infertility and procreation medicalization. This defensive mechanism prevents awareness and emotional expression and reduced fantasies. This mechanism could mimic a good acceptance of the situation or even indifference. But it impacts the couple’s relationship. The female partner could indeed feel like she is “the only one suffering”. It can also affect the medical MAR attempt as if everything “went well” for him. Among both men and women difficulty in partner communication, having difficulty in talking about emotional consequence of infertility and treatment process with the partner is indeed a significant predictor of stress [22]. According to other authors [23], it can have direct or indirect effects on MAR outcomes: fertility problem stress arising in the personal and marital domain show greater association with treatment outcome, women who report mare marital distress require more indeed treatment cycles to conceive.

To summarize, male infertility suffering exists and there are different ways to cope with the trauma of procreation project failure. Here, illustrated by the cases of two infertile patients, we reported our psychodynamic experience. These case reports will help us to understand the benefit of psychoanalytic care in infertile men.

## Material and methods

This study was part of a broader study which was approved by an independent ethics committee (number 0132, 21 November 2002) and was registered with the French health authorities.

### Patients

Here we reported the case of two infertile patients who underwent MAR at Poissy hospital (France).

### Methods

Here, our aim was not to compare different psychological methods. We described our own method, the psycho-analytic one. For these two patients, we used one to one in-depth interviews and a psychoanalytic/psychodynamic approach as previously described for infertile men. Three key tools of a successful psychodynamic approach are: the provision of insight through the use of transference, a focus on patient affect, and the therapist’s attention to aspects of the therapeutic alliance [24, 25].

### Psychoanalytic/dynamic theoretical framework

Firstly, let us first consider what happens for infertile women and infertile couples. For a woman, it is an objectal and narcissistic wound. Beside the disappointment of not receiving this child secretly desired from the father since childhood, she feels the pain of not being able to reconnect with the long-lost mother of life’s beginnings, through her own motherhood. Hence, a child will always be missing to compensate for all the sorrows and pain of life [26]. The aching of this incompleteness holds the largest place. Added to this pain is the feeling of being excluded from the process that makes other women of the same generation become mothers in the footsteps of their own mothers. Infertile women can feel infantilized, sent back to a little girl status: someone who was dependent on her mother. This may reactivate her childhood conflict with her.

From the point of view of couples, a “sterile sexuality” (which does not result in the birth of a child) challenges the link built between sexuality and procreation at the Oedipus stage: “sterile sexuality challenges the Oedipus complex position; it consequently threatens personal identity and entails intense suffering” [5, 27]. The personal identity built during childhood is challenged: sexual identity as well as affiliation to the lineage. Furthermore, the extension of the possibilities offered through MAR has consequences. The couple and their doctor get indeed into a spiraling offer of care bypassing what is at stake in natural procreation As the promise of a conception outside sexuality is made, the pain of a “sterile sexuality” is set aside preventing the elaboration of the trauma [28]. Thus it gives space for the denial of this suffering.

In men, the suffering caused by infertility can have several aspects. It can display a narcissistic dimension when it challenges his virility since fertility and virility are intertwined in people’s mind as mentioned before [4, 29, 30]. It can display an objectal dimension (object-libido), turned toward others, be it a spouse “presenting a partner with a child” or a parent “presenting his parents with a grandchild”, as a token of eternity or a gift to repair the odds of life [31, 32]: here it is the suffering of infertility from a generational point of view that has to be accounted for both men and women. It is, according to Bydlowsky, linked to the inability to settle the debt of life toward their parents, the impossibility to repay a trans-generational debt. Despite a long-lasting treatment of male infertility, male infertility remains surrounded with secrecy more often than in female infertility. Announcing male sterility remains difficult to confess, especially to the father of the infertile man. Indeed, when seen through unconscious Oedipus complex, the impossibility for a man to become a father can mean the realization of the threat of castration that was feared during childhood. This is a result of incestuous desires of the little boy for his mother, getting rid of the father to take his place with his mother. But it can also have an identity dimension which cannot be soothed if doctors propose a gamete donation too early. This proposal can even paradoxically block the mourning process of infertility.

Two clinical cases will illustrate these aspects of the suffering of male infertility. The choice to present different situations of partial and total infertility is intentional. The aim is to show that the pain caused by a partial primary infertility is not weaker than the pain linked to definitive sterility. Undeniably the fantasies and anxieties revived are similar, they proceed from the vicissitudes of the psycho affective development of the child these men once were.

## Results: the two cases reports

### Bertrand: the outstanding debt

For Bertrand the diagnosis of infertility was devastating. He compared it with a serious illness like cancer, even though the couple was offered the possibility of an ICSI (intracytoplasmic sperm injection) attempt. He felt very depressed and is recovering slowly “I don’t know how I can get over all that. I am still, how can I say it, digesting the information and I don’t know if this digestion is proceeding slowly, so I cannot think about tomorrow, I must cope with what I am doing (accepting my infertility) from day to day”. His story is particular: Anonymous childbirth*,* he is the adopted son of a couple who turned to adoption because of the husband’s infertility. The relationship with his own father had been completely severed after his parents divorced when he was still a child. He does not see his mother either.

Abandoned by his own father, Bertrand’s father struggled with his role as a father, he acted more like a buddy, father and son had an overly close relationship avoiding all conflicts : “we were close with my father whose education was that of father and son but not in a classic sense …a relationship in which we stuck together and we were attentive to one another …a very tender relationship because his own father had disappeared so soon, well he had left and …a relationship to an only son so much desired, the ninth wonder in the world. He had no authority whatsoever and I am afraid to recreate the same attitude toward my children, that is to say having a very complacent attitude to avoid conflicts, which is not the best way to act with children”. When Bertrand ran away when he was a teenager, his father did not scold him, did not say anything when the young man disappeared for months. He did not try to contact him. “He did not worry really”, when as a matter of fact Bertrand was living close to his parents. Bertrand did suffer from the situation “it would have been easy to locate me” says this former child who had already been abandoned before his adoption.

### George: a flickering identity

During the first interview, we met a man defeated by the news of his infertility. He was the youngest child of its family. The parents were idealized, particularly his father, a genuine *Pater Familias* whom he has always wanted to look like. This idealization hided in fact an Oedipus conflict and an opposition to the family embodiment. George’s schooling trajectory was chaotic contrary to his brothers who completed higher education. He chose a job which was not up to the family expectations. He used to avoid all conflicts due to father’s authority relationship. He did not feel quite like a man, more as an eternal adolescent; he has always fantasized about becoming a man the day he becomes a father.

The diagnosis of infertility came as a terrible shock that has not been softened even after months passed and though there is the prospect of a gamete donation. His identity markers have been shaken: being a man/a woman, belonging to the community of fathers, being a husband, being a father… If he does not become a father, can he be a man? Wonder he. He will never belong to the community of fathers like his father and brothers, to the community of men; he will be in an unacceptable in between “the childless uncle”, forever a teenager. He can’t accept becoming a father through a gamete donation because this child will always remind him and others that he is sterile. He fears he won’t be able to love that child, won’t be able to get involved as a father, to take on the father role while this child will remind him of his exclusion from the Men/Fathers world. At the same time, he can’t refuse because he is sure that he would lose his wife and with her this other identity support: being “the husband of someone”. He does not have the time needed to grieve because of his wife’s age which requires that the donation process be sped up. His suffering is extreme. He can’t talk to anyone about it. He can’t get support from his close relations because if he talked about it the reality of his sterility it would become definite. For the moment, he lets time go by, without talking about his doubts, letting things happen, waiting for a relief that does not come, clinging to the very weak hope that he can become a father naturally.

## Discussion

For Bertrand there is a weakening of the symbolic filiations link, the one that provides ties but also makes the difference (one is son of, brother of, but one is not his father or brother). Thus it is the narcissistic, imaginary dimension of this link, the repetition of the same that takes precedence. In an imaginary unconscious identification (and not a symbolic one) to his father, a “body to body” relationship, he will be sterile as his father was and is. Like other infertile men, by means of his sterility he avoids to pay back his life debt to this adoptive father. However, he also suffers from that… He is unable to give him a grandchild. He also cannot mend the generational thread that has been damaged by “filiations break” at his own level and at his father’s level. Bertrand is burdened with the narcissistic wound of his adoptive father (having been abandoned by his father, being sterile) and can only work at protecting him avoiding “the murder of the father task” implied through fatherhood. Symbolically, killing one’s father when one becomes in turn a father. He supports his father. He accumulates names but cannot consider any of them as own. The identity dimension can be stressed in the questioning of generations “Who am I? Who have so much difficulty with genealogy?” he can wonder. He will have to be given time to accept and follow through the ICSI proposal made to the couple. If the result is positive, he will have to face the possibility to become a father and symbolically kill his own father. If it is negative he won’t be able to pay back his “life debt” toward him.

In George, besides the castrating narcissistic wound (sterility being equivalent to the actual performance in his body of the anguish of imaginary castration of the Oedipus complex) it is the identity dimension for the sterility suffering that is expressed. “Who am I? a man or a woman? a grown-up or forever a teenager? What is my place in my parentage?” Here are the questions he sadly asked. It seems that for the moment the donation project stops the process of elaboration and sublimation of the loss of his procreative capacity through a response that comes too soon. Even if he wishes to build a family, he was afraid of not being able to consider the child who would be born from a donation as his own. He could reject him as this child would remind him his sterility. Hence, psychotherapeutic interviews helped him to reintroduce a dialogue with his wife, a dialogue that had been interrupted.

Globally, which support could be proposed? These two cases in which the psychological suffering of men confronted with their infertility (whether total or partial) is openly expressed are rather exceptional when considering the expression of this suffering. Most of the time, psychological consultations are asked for by women. If men suffer, they don’t talk about it and rarely seek a psychological consultation [10]. However, in our experience, they more often come with their partner (about 15% of cases) to search for help they cannot express directly for themselves. We have mentioned the particular type of defense that men very often set up to fight the profound helplessness they experience and the anxiety reactivated by psychological wounds, whether narcissistic, castratic or identity related. They consist of repressing the affects, a denial or a trivialization of this suffering for the benefit of the consideration of their partner’s. Often we noticed that they refuse to talk about it to their wife, their relatives or their friends. We have seen that the defense mechanism is close to psychosomatic functioning, and even if it protects them partially, it tends to halt the task of elaborating on the infertility wound and sometimes the mourning of the procreative capacity which would allow a real clearing, a genuine sublimation. In those instances providing care is difficult because it questions the possibility to encourage those men to open the wounds they so carefully protect. When they accept the risk, a genuine elaboration task can take place. The interest of these cases report was not to compare any therapy with another one (cognitive, psychoanalytic, support…). On the contrary, we believe it was important to draw attention to the benefit of such psychoanalytical/psychodynamic interviews in somatic field, as it was previously mentioned [24, 25]. We already have experimented this in our own research on male infertility [20]. Indeed, for patients undergoing MAR, the fact of talking of their personal history was reported to improve the success of the MAR attempt. As previously reported, we hypothesized that the improvement of communication between the two members of the couple had also a positive impact on the MAR outcomes [33]. So, listen to these infertile men is primordial. Any protagonist of the MAR process can listen to them. These moments of emotional opening may occur with one or the other therapist (psychologist, gynaecologist, nurse…). An empathetic attention to infertile men may give a way to express this suffering and thus allow the beginning of a psychoanalytic approach which is necessary in infertility and especially for infertile men who do not easily express their suffering.

## Conclusion

We can say that it is crucial that MAR actors allow themselves to be touched and made aware by the particular stakes brought through each procreation project and its difficulties. Not being fooled by the apparent absence of difficulty, trying to put words on what empathy can detect notwithstanding the screen of silence, giving a listening time that the infertile man does not allow to himself can open a space for words and sometimes start the mourning of his fertility. The two clinical case reports were used here to (i) illustrate all these aspects of infertility suffering, (ii) to evidence the difficulty for infertile men to speak about their infertility and (iii) underlie the importance for professional of medical assisted reproduction to be attentive to this suffering that many men keep silent. An empathetic attention to infertile men may give a way to express this suffering and thus allow the beginning of a psychoanalytic approach which is necessary in infertility and especially for infertile men who do not easily express their suffering.

## Consent

Written informed consent was obtained from the patient for the publication of this report and any accompanying images.

## Abbreviations

MAR: Medical assisted reproduction.
